# Physical exercise in haemodialysis patients: which type of exercise is more convenient?

**DOI:** 10.1093/ckj/sfae165

**Published:** 2024-06-10

**Authors:** Martin Halle, Fabio Manfredini, Jürgen Floege, Carmine Zoccali

**Affiliations:** Department of Preventive Sports Medicine and Sports Cardiology, TUM School of Medicine and Health, TUM University hospital ‘Klinikum rechts der Isar’, Technical University of Munich, Munich, Germany; DZHK (Deutsches Zentrum für Herz-Kreislauf-Forschung), partner site Munich, Munich Heart Alliance, Munich, Germany; Department of Neuroscience and Rehabilitation, University of Ferrara, Ferrara, Italy; Division of Nephrology, University Hospital, Rheinisch-Westfälische Technische Hochschule (RWTH) Aachen, Aachen, Germany; Renal Research Institute, New York, USA; Institute of Molecular Biology and Genetics (Biogem), Ariano Irpino, Italy; Associazione Ipertensione Nefrologia Trapianto Renale (IPNET), c/o Nefrologia, Grande Ospedale Metropolitano, Reggio Calabria, Italy

**Keywords:** home-based exercise, intrahemodialysis exercise, kidney failure, physical exercise

## Abstract

Randomized intra-haemodialysis and home-based exercise trials have demonstrated similar efficacy in improving physical performance, particularly in increasing walking distance. During dialysis sessions, patients can engage in structured, supervised activities such as cycling or resistance exercises, ensuring safety and immediate feedback from healthcare professionals. This structured nature can significantly enhance adherence, making exercise a regular part of the patient's treatment schedule. Home-based exercise offers flexibility and convenience. Patients can incorporate activities like walking, stretching or using resistance bands into their daily lives. This flexibility allows patients to exercise at their own pace and according to their preferences, fostering independence and self-management. By continuing physical activity at home, patients can maintain continuity in their exercise regimen, which is crucial for long-term health benefits. Combining both intra-haemodialysis and home-based exercises has the potential to improve overall adherence to exercise programs. Strategies such as patient education, customized plans, monitoring and feedback, and support systems can help combine these two exercise types. By integrating these two modalities, healthcare providers can create a comprehensive and balanced exercise regimen that enhances adherence, promotes independence and maximizes health benefits for dialysis patients, fostering long-term health and well-being through sustained physical activity. However, this dual approach, which caters to both the need for medical supervision and the desire for personal autonomy, has yet to be tested in randomized trials.

## INTRODUCTION

Physical activity is a potent modifier of numerous aspects of health, enhancing cardiorespiratory fitness, bolstering the strength and integrity of muscles and bones, and fostering mental and cognitive wellness. The Centers for Disease Control and Prevention recommends physical activity as a preventative measure against a spectrum of conditions, such as obesity, type 2 diabetes, cardiovascular diseases, various cancers, and mental health disorders like depression and anxiety, as well as cognitive decline and dementia [1].

In the context of chronic kidney disease, particularly for patients undergoing dialysis, inactivity is a pervasive issue. This population frequently experiences a sedentary lifestyle, fatigue, depression and cognitive dysfunction, diminished physical capabilities and a compromised quality of life. The lack of adequate resources and infrastructure to facilitate exercise programs, especially in areas with limited means, presents significant challenges in promoting physical activity among those receiving dialysis.

The exercise modalities that can be applied in dialysis patients range from aerobic activities like cycling during haemodialysis or walking at home to resistance exercises such as weightlifting or using resistance bands—either at the dialysis facility or at home—or a combination of aerobic and resistance training. Cycling is the most common aerobic exercise within in-centre programs [2]. No single form of intradialytic or home-based exercises stood out as superior to others in improving the studied outcomes, including physical performance and quality of life. Importantly, regardless of the setting, exercise programmes that lasted longer than 12 weeks and those of moderate to vigorous intensity were more effective at enhancing functional capacity than shorter or less intense regimens [2]. In essence, exercise programmes confer benefits to those on dialysis wherever they are carried out.

Long-term observation in the frame of the EXCITE (EXerCise Introduction To Enhance performance in dialysis patients) study [3], a randomized trial that showed a beneficial effect on physical performance of a home-based walking exercise in haemodialysis patients, documented that an increase in distance walked during a 6-minute walk test (6MWT) correlates with a significant reduction in the long-term risk for a composite endpoint, including death from any cause, cardiovascular events and hospitalizations [4]. More recently, the DiaTT (Dialysis Training Therapy), the largest trial testing an exercise intervention in haemodialysis patients so far [5], showed that a thrice-weekly supervised endurance and resistance exercise programme during haemodialysis improved the 60-second sit-to-stand test (STS60)—a measure of lower body strength elected as the primary study outcome—and the walking performance, assessed by the 6MWT, a secondary endpoint, over a 1-year treatment.

Given the difference in logistics and cost of the two interventions, *CKJ* invited two leading authors of the studies above, Dr Martin Halle [5] and Dr Fabio Manfredini [3], to debate which exercise should be preferentially applied in the haemodialysis population.

## PRO: INTRA-HAEMODIALYSIS EXERCISE

**Figure 1:**
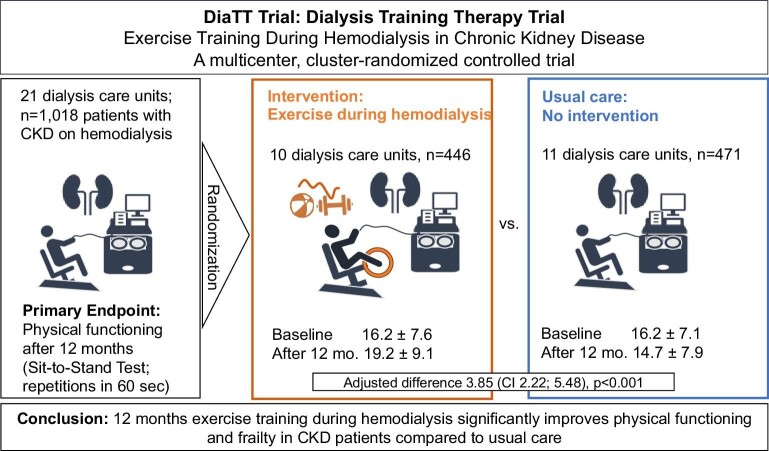
Study design and results of the DiaTT trial.

The contender, Dr Halle, remarks that implementing exercise into routine practice has so far failed. He emphasizes that the DiaTT is one of the largest randomized controlled trials on exercise in medicine. The DiaTT trial has assessed the feasibility and efficacy of intradialytic exercise training in a large population of >1000 kidney failure patients, who were randomized either to intradialytic exercise or to usual care in a cluster-randomized setting including 21 dialysis units in Germany. After 12 months of intervention, patients performing combined endurance and resistance exercise for 30 min per dialysis session significantly improved the STS60, which measures how well patients can rise from a sitting to a standing position, an ability relevant for independent living, an improved timed-up and go test (TUG), assessing leg strength in combination with coordination, and in the distance walked during the 6MWT, a measure of aerobic endurance capacity. The latter improved on average by 37.5 m, which is clinically important in chronic disease (Fig. 1). These findings were accompanied by a significant reduction in annual hospital admissions and almost halving days spent in hospital in this multimorbid patient population.

Importantly, a large and broad proportion of patients (57% of all dialysis patients) of participating dialysis centres were included in the study. Almost 60% were older than 65 years, and one-third had coronary heart disease, heart failure or diabetes. Moreover, the representativeness of the DiaTT patient population for the general German kidney failure patient population was confirmed by comparing DiaTT patients with >10 000 dialysis patients from German health insurance [5].

Intradialytic exercise is training by cycling on a bed ergometer and performing resistance exercises while lying supine. Time during haemodialysis, e.g. 4–5 h, is used for exercise instead of quietly lying during haemodialysis. The former has clear advantages over the latter as the dialysis exercise setting is supervised, which (i) allows patients with comorbidities and frailty to participate in the exercise program, (ii) increases adherence by initiating exercise with each haemodialysis session and (iii) improves the efficacy of haemodialysis, as observed when patients increase their cardiac output when cycling during haemodialysis. Dr Halle notes that home-based training has been proposed as an alternative and less cost-intensive intervention to intradialytic exercise training. However, he remarks that results have only been obtained from shorter interventions in smaller, selected cohorts [6]. Even though Dr Halle accepts that exercise at home is an option, he adds that his group experienced difficulties in adherence in this setting. When supervised exercise within the DiaTT trial had to be replaced by home-based training during the COVID-19 pandemic, the German investigators observed lower adherence rates than during intradialytic training phases.

Dr Halle emphasizes that by including a representative real-world kidney failure population undergoing regular haemodialysis, the DiaTT trial has overcome widespread skepticism by dialysis staff towards intradialytic exercise, questioning the suitability of exercise for old and frail patients with multiple comorbidities by proving feasibility and safety. The positive patients’ feedback (‘I am not going to have haemodialysis, but instead I am attending my exercise dialysis work-out’) and broad clinical benefits on frailty parameters and hospitalization support the implementation of intradialytic exercise into routine health care. He concludes that, because the economic costs of supervised exercise intervention, e.g. trainers and equipment, are less than the amount of money saved by a reduction in hospital days, the German joint federal committee has recommended that health insurance reimburse intradialytic exercise.

## PRO: HOME EXERCISE

The contender , Dr Fabio Manfredini, stresses that any option for delivering training to a broader range of eligible sedentary individuals is worthy of interest given the hazard of physical inactivity and the potential benefits of exercise. He specifies that the home-based approach should not refer to a simple increase in spontaneous activity but rather to executing home training sessions that are planned and organized according to the components of the Frequency, Intensity, Time and Type (FITT) principle of exercise prescription. Unfortunately, for kidney failure patients, as for many diseased and healthy individuals, the willingness to adhere to exercise programmes clashes with multiple barriers that come between patient intention and action [7]. From this perspective, home-based programmes overcome some organizational, physical and psychological obstacles typical of supervised hospital-based programmes. They do not require the constant availability of dedicated equipment and staff or the need to transport the patient to the hospital in case of an inter-dialysis programme or the exercise execution during dialysis. This aspect concerns patients for the fear of aggravating fatigue or health status. Home-based programmes allow individuals to exercise regularly at home on non-dialysis days, positively impacting their lifestyle. This approach may generate the habit of maintaining a daily amount of exercise, self-management of one's physical condition through non-pharmacological intervention and the belief that the hospital is not the only place conducive to improving one's health status. Further elements supporting the home-based exercise approach include the shift of care activities toward the home, the containment of healthcare costs, and the exploitation of valuable technologies such as wearable devices, remote connections and shared data management.

Dr Manfredini underlines that home-based exercise is a generally effective intervention for kidney failure patients with different exercise modes. He quotes a recent systematic review and meta-analysis reporting significant improvements in physical performance following home-based programmes lasting 3–6 months [7]. Among the studies in the meta-analysis, the EXCITE trial [8] was the most extensive study testing a home-based intervention that included 38% of the patients in the systematic review. As underlined also by Dr Halle, this trial documented that a 6-month home walking training programme increased walking performance and improved the STS60 test, a test that primarily measures the strength of the lower body muscles, including the quadriceps, hamstrings and gluteal muscles, which are essential for performing daily activities such as walking, climbing stairs and getting up from a seated position. Two dimensions of quality of life improved significantly during the EXCITE trial [3]. Furthermore, an observational extension of the same trial to 2 years showed that the 6-month intervention reduced the risk of hospitalization in patients originally allocated in the active arm of the trial [4].

**Figure 2:**
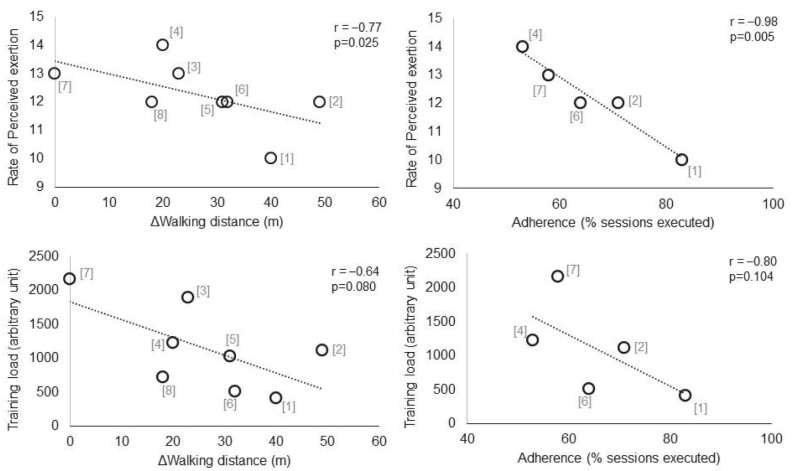
Rank correlation between mobility at the end of the home-based programme and the rate of perceived exertion (left upper panel) and the training load (left bottom panel). Rank correlation between percentage of adherence (right upper panel) and rate of perceived exertion or training load (right bottom panel). Numbers in the graph refer to source studies included in the Battaglia meta-analysis (reference [7]). In detail: [1] Manfredini *et al. J Am Soc Nephrol* 2017;28:1259–68; [2] Koh *et al. Am J Kidney Dis* 2010;55:88–99; [3] Myers *et al. Kidney Blood Press Res* 2021;46:196–206; [4] Ortega-Pérez de Villar *et al. Sci Rep* 2020;10:8302; [5] Watanabe *et al. BMC Nephrol* 2021;22:98; [6] Uchiyama *et al. Ther Apher Dial* 2020;24:668–76; [7] Bohm *et al. Nephrol Dial Transplant* 2014;29:1947–55; [8] Perez-Dominguez *et al. Eur J Phys Rehabil Med* 2021;57:994–1001.

The EXCITE trial can be seen as an evolution of a previous non-randomized study with a similar programme that documented positive effects on functional and clinical long-term outcomes in patients with peripheral arteriopathy [8]. These effects were obtained with minimal instrumentation and staff participation, according to a low-cost model of daily care offered to patients with medium-severe arteriopathy and with a low-intensity approach through an original setting and design. In the previously quoted meta-analysis by Battaglia *et al*. [7], a measure of mobility was reported in 8 out of 12 studies. A separate analysis of these studies showed that the intensity (Borg scale) and the total training load prescribed, based on the FITT principles, were inversely correlated with the within-group functional changes (Fig. 2). Adherence, reported only in five studies, also showed an inverse association with the same variables.

A prudent prescription of the training load in home-based exercise programmes may ensure safe execution with minimal risk of major or minor adverse events and favour broad inclusion, particularly for patients with comorbidities and restricted mobility, who are often excluded from exercise programs. Furthermore, the home programmes must be sufficient in density (two to three times a week) and duration (12–24 weeks). Using technologies and wearable devices may enable precise and safe execution, monitoring of training adherence and large data collection. Training should target mobility and balance and aim to reduce the risk of falls during daily activities. After formal testing in clinical trials, home-based exercise programmes should be implemented in real life. Effective interventions in the literature derive from trials where enrollment criteria are strictly defined and where the number of dedicated operators and available equipment were possible because of dedicated funding, often lacking in daily clinical activity. On the other hand, organizational models should respond, as is the case for technologies, to a degree of maturity and readiness to ‘enter the market’ based on validation trajectories regarding feasibility, large-scale management, adherence and possibly economic sustainability. In closing, Dr Manfredini suggests that an alternative possibility is to organize community-based programmes inside gym clubs to combine aerobic and resistance training under the supervision of physiotherapists, kinesiologists and exercise specialists [2]. To ensure broader adherence and a patient-centred approach, effective hybrid solutions involving both hospital and home-based interventions might be considered to allow the person's preferences on location and mode of exercise execution.

## THE MODERATORS’ VIEW

The moderators believe that the choice between intra-haemodialysis and home-based exercise hinges significantly on available resources and individual incentives. The pluses and the minuses of these exercise types are schematized in Table 1. Both have demonstrated comparable efficacy in enhancing physical performance, particularly in increasing walking distance, a critical measure of functional capacity. Intra-haemodialysis exercise offers the advantage of a supervised environment, ensuring patient safety and potentially improving adherence due to the structured setting and constant reminders. However, it requires investment in equipment and trained personnel, making it more resource-intensive. This format, possible only in countries without stringent financial limitations to healthcare, is particularly beneficial for patients who prefer or need the reassurance of medical supervision during physical activity. Conversely, home-based exercise provides greater flexibility and cost-effectiveness, allowing patients to incorporate physical activity into their daily routines without requiring specialized equipment or supervision. This can promote independence and self-management, although it may come with challenges in maintaining consistent adherence and ensuring safety, especially for those with severe comorbidities.

**Table 1:** Advantages and disadvantages of intra-haemodialysis and home-based exercise in haemodialysis patients.

Intra-haemodialysis exercise
Advantages
• Supervised setting: intra-haemodialysis exercise is conducted under medical supervision, ensuring safety, especially for patients with comorbidities and frailty
• High adherence: regular exercise during dialysis sessions can increase adherence since it becomes part of the patient's routine
• Improved dialysis efficiency: exercise during dialysis can enhance haemodialysis efficacy by increasing cardiac output
• Proven benefits: the largest trial performed so far, the DiaTT trial, demonstrated significant improvements in physical performance measures (e.g. STS60, 6MWT) and reduced hospital admissions
Disadvantages
• Cost: requires investment in equipment (e.g. bed ergometers) and trained staff to supervise the exercise sessions
• Logistics: implementing such programmes can be logistically challenging, needing coordination and space within dialysis centres
Home-based exercise
Advantages
• Flexibility: patients can exercise at their convenience, which can help integrate physical activity into their daily routine
• Cost-effective: home-based programmes typically require minimal equipment and no need for dedicated staff, reducing costs
• Promotes independence: encourages self-management and can foster a habit of regular physical activity beyond dialysis sessions
Disadvantages
• Adherence issues: without supervision, patients may struggle to maintain consistent exercise routines
• Safety concerns: patients with severe comorbidities or mobility issues may face higher risks of injury without professional guidance
• Variable outcomes: the effectiveness can vary widely depending on the patient's motivation and ability to follow the prescribed exercise regimen

The choice between the two treatments is often dictated by available resources and individual incentives. However, considering these two forms of exercise as mutually exclusive options overlooks the potential benefits of a synergistic approach that combines elements of both. Integrating intra-haemodialysis exercise with home-based exercise can create a balanced and comprehensive exercise regimen. Patients can engage in structured, supervised activities such as cycling or resistance exercises during dialysis sessions. This ensures safety, allows for immediate feedback from healthcare professionals and helps patients establish a routine. The structured nature of intra-haemodialysis exercise can significantly enhance adherence as it becomes a regular part of the patient's treatment schedule. Outside of dialysis sessions, home-based exercise offers flexibility and convenience. Patients can incorporate activities like walking, stretching or using resistance bands into their daily lives. This flexibility allows patients to exercise at their own pace and according to their personal preferences, fostering a sense of independence and self-management. By continuing physical activity at home, patients can maintain continuity in their exercise regimen, which is crucial for long-term health benefits. This dual approach caters to both the need for medical supervision and the desire for personal autonomy, addressing dialysis patients’ diverse needs and preferences. So far, there is no evidence that physical activity in dialysis patients can be ‘over-dosed’ if the two modalities are combined.

To effectively combine intra-haemodialysis and home-based exercises, several strategies can be employed. First, educate patients on the benefits of both types of exercises and provide training on how to perform home-based exercises safely. Instructional sessions during dialysis or take-home materials and virtual resources can be utilized to ensure patients are well-informed and confident in their ability to exercise at home. Individualized exercise plans that incorporate both intra-haemodialysis and home-based activities can be construed. Healthcare providers can assess patients’ capabilities, preferences and goals to create a balanced regimen that maximizes benefits. Fitness trackers or mobile apps to monitor patients’ activity levels at home may be adopted. Regular check-ins during dialysis sessions provide opportunities to review progress, address concerns and adjust the exercise plan as needed.

Encouraging the formation of support groups or buddy systems among patients to foster a sense of community and mutual encouragement should be pursued. Sharing experiences and progress can enhance motivation and adherence to the exercise regimen.

In conclusion, the choice of exercise type for dialysis patients should be guided by available resources and individual preferences. Both intra-haemodialysis and home-based exercises have been shown to offer significant health benefits, particularly in improving walking distance. By integrating these two modalities, healthcare providers can create a comprehensive and balanced exercise regimen that enhances adherence, promotes independence and maximizes the overall health benefits for dialysis patients. This synergistic approach not only addresses the immediate needs of patients during dialysis, but also fosters long-term health and well-being through sustained physical activity at home.
